
JOURNAL INFORMATION
==============================
Journal ID: Wirtschaftsdienst

Wirtschaftsdienst

EISSN: 1613-978X

ARTICLE INFORMATION
==============================
PMCID: PMC9307436
DOI: 10.1007/s10273-022-3239-8
Article ID: 3239
Article version: 1
Subjects: Zeitgespräch

Mehr Selbstdisziplin macht Politikberatung besser

Self-Discipline Makes Policy Advice Better

Wagner Gert G. gwagner@diw.de

für Verbraucherfragen, Friedrichstraße 191, 10117 Berlin, Deutschland
Electronic publication date: 2022 Jul 23
Print publication date: 2022
Volume: 102
Issue: 7
First page: 518
Last page: 521
Copyright: © Der/die Autor:in 2022, korrigierte Publikation 2022
License: Open Access: Dieser Artikel wird unter der Creative Commons Namensnennung 4.0 International Lizenz veröffentlicht (https://creativecommons.org/licenses/by/4.0/deed.de).
Open Access wird durch die ZBW — Leibniz-Informationszentrum Wirtschaft gefördert.
License URL: https://creativecommons.org/licenses/by/4.0/

Keywords: A11, A13, A23, D83
issue-copyright-statement: © The Author(s) 2022

==============================

Der Wirtschaftsdienst hat in verdienstvoller Weise immer wieder dem Thema Politikberatung Raum gegeben und der Autor dieses Beitrags hat sich daran beteiligt (insbesondere Hoffmann und Wagner, 1998; Wagner, 2006, 2011; Fratzscher und Wagner, 2013 sowie Haucap et al., 2015). Warum also ein weiterer Beitrag zu diesem Thema? Es gibt in der Tat aus verschiedenen Gründen etwas Neues zu sagen und zu diskutieren; dieser Beitrag wird insbesondere auf die Bedeutung der Digitalisierung der Wissenschaftskommunikation eingehen und dabei einige wesentliche Befunde und Empfehlungen einer Interdisziplinären Arbeitsgruppe (IAG) der Berlin-Brandenburgischen Akademie der Wissenschaft (Weingart et al., 2022; Wormer, 2022) und der vom BMBF organisierten #FactoryWissKomm (FactoryWissKomm, 2021) vorstellen und kurz diskutieren.[1]

Der Begriff Politikberatung ist eng mit den Wirtschaftswissenschaften verbunden – andere Disziplinen sprechen heutzutage eher von Wissenschaftskommunikation, die den Begriff Wissenstransfer abgelöst hat. Gemeint ist nicht die Beratung der Politik im Allgemeinen, insbesondere nicht die Beratung durch Nicht-Wissenschaftler:innen (z. B. Leif, 2015), sondern die Beratung der Politik durch forschende Wissenschaftler:innen. Zur wirtschaftswissenschaftlichen Politikberatung gibt es eine differenzierte Literatur, die jedoch weitgehend unverbunden mit der Literatur (und der dahinterliegenden Forschungslandschaft) zur „Wissenschaftskommunikation“ ist, die sich ursprünglich nur mit den Naturwissenschaften beschäftigt hat. Und beide Diskussionsstränge sind kaum verbunden mit der Figur des in den Geistes- und Sozialwissenschaften seit Jahrzehnten eine große Rolle spielenden Öffentlichen Intellektuellen (z. B. Bock, 2011); einer Rolle, die – so wird im vorliegenden Beitrag diskutiert – nun viele Wissenschaftler:innen mit niedrigen Zugangshürden digital spielen können.[2] Hier kann nicht im Einzelnen auf die einschlägige kommunikationswissenschaftliche Literatur eingegangen werden, es sei aber darauf hingewiesen, dass sich die (selektive) Auseinandersetzung mit dieser Literatur auch im Hinblick auf Politikberatung lohnen könnte. Einen aktuellen Überblick geben Weingart et al. (2022).

Hier explizit erwähnenswert ist der Hinweis (Weingart et al., 2022): „Anlässlich einer forschungsstrategischen (ersten) Aufarbeitung der Covid-19-Krise macht der Wissenschaftsrat (2021) eine begriffliche Unterscheidung zwischen Politikberatung und Transfer (S. 8), ohne jedoch auf die Unterschiede näher einzugehen. Politikberatung und Wissenschaftskommunikation fasst er auf S. 10 unter dem Titel „Zusammenwirken mit Politik und Öffentlichkeit“ zusammen. Man kann daraus schließen, dass im Verständnis des Wissenschaftsrats Politikberatung auch Wissenschaftskommunikation ist, die sich an einen speziellen Adressatenkreis richtet („die” Politik). Müller-Jung (2021) bringt es in einem Kommentar in der Frankfurter Allgemeinen Sonntagszeitung auf den Punkt: „Politikberatung durch Fachleute ist Wissenschaftskommunikation.“ Ähnlich wird auch zwischen Politikberatung und Gesellschaftsberatung unterschieden (etwa BBAW, 2008).

## Neuere Entwicklungen

Im Folgenden wird es darum gehen, was wir für die Politikberatung aus einer Reihe von Stellungnahmen der deutschen Wissenschaftsorganisationen für die Organisation

1 Der Autor war Mitglied der Interdisziplinären Arbeitsgruppe (IAG) „Implikationen der Digitalisierung für die Qualität der Wissenschaftskommunikation“ der Berlin-Brandenburgischen Akademie der Wissenschaften und der vom BMBF organisierten „#FactoryWissKomm“. Wie z. B. Christian Drosten ist auch der Autor kein ausgewiesener Kommunikationsfachwissenschaftler, äußert sich aber trotzdem zur Wissenschaftskommunikation.

2 Auf die auf Wertungen abzielenden „Orientierungswissenschaften“, die Gelehrte und Öffentliche Intellektuelle anbieten, kann hier nicht näher eingegangen werden (Wagner, 2005). Es sei zumindest daran erinnert, dass das Feuilleton ursprünglich ein Abschnitt zu „den gelehrten Sachen“ (in der Vossischen Zeitung war (bis 1911 unter anderen Namen erschienen, der seit 1740 zeitweise die „gelehrten Sachen“ explizit nannte; Bender, 1972). Von 1751 bis 1755 war Gotthold Ephraim Lessing der verantwortliche Redakteur, der das Feuilleton auf eine in Berlin noch nicht gekannte Höhe“ hob (Bender, 1972). Zu diesem Feuilleton gehörten ausdrücklich „Wissenschaftsnotizen“, wozu Mitte des 19. Jahrhunderts zeitweise eine „wissenschaftliche Beilage“ zur Sonntagsausgabe gehörte (Bender, 1972).

einer qualitativ guten Wissenschaftskommunikation und damit auch einer entsprechenden Politikberatung lernen können. Die Nationale Akademie Leopoldina, zusammen mit der Deutsche Akademie der Technikwissenschaften (acatech) und der Union der deutschen Akademien der Wissenschaften hat bereits 2014 eine Stellungnahme „Zur Gestaltung der Kommunikation zwischen Wissenschaft, Öffentlichkeit und den Medien" veröffentlicht sowie 2017 die Analysen und Empfehlungen zu Social Media und digitaler Wissenschaftskommunikation (Leopoldina et al. 2014, 2017). Wie Weingart et al. (2022) zusammenfassen, wurde bereits 2014 zur Kommunikation zwischen Wissenschaft, Öffentlichkeit und den Medien u. a. empfohlen,

- die Kommunikationsstrategien wissenschaftlicher Einrichtungen im Hinblick auf Qualitätsstandards und „wissenschaftliche Redlichkeit" zu überarbeiten. (Empfehlung 1)
- „… ein übergreifendes Qualitätslabel für vertrauenswürdige Wissenschaftskommunikation zur Auszeichnung institutioneller Pressearbeit unter den genannten Kriterien einzuführen." (Empfehlung 2)
- „Nicht durch Daten bzw. Evidenzen gedeckte Übertreibung von Forschungsergebnissen gegenüber den Medien (Hype) soll als Verstoß gegen gute wissenschaftliche Praxis gelten und entsprechend sanktioniert werden." (Empfehlung 3)

Weingart et al. (2022) weisen auch auf die Stellungnahme aus 2017 hin, die die digitale Wissenschaftskommunikation im Fokus hat. Dort heißt es: „PR- und Presseabteilungen bzw. Abteilungen zur vermittelnden Wissenschaftskommunikation [müssen] – im Sinne des Transparenzgebots – von nach Regeln der Werbung handelnden Marketingabteilungen (wieder) klar getrennt sein – vergleichbar der strukturellen Trennung von Redaktion und Anzeigenabteilung in journalistischen Medienhäusern." Ganz konkret: „Bei Universitäten könnten solche primär der Wissenschaftskommunikation im ursprünglichen Sinne und weniger der Reputationskommunikation verpflichteten Stellen nicht der Leitung der Institution, sondern zum Beispiel dem Senat oder vergleichbaren Aufsichtsgremien unterstellt sein" (Weingart et al., 2022, 53).

Der Wissenschaftsrat (2021) spricht jüngst explizit die bei der Verwendung der digitalen Medien entstehenden Qualitätsprobleme an: „Wissenschaftlerinnen und Wissenschaftler müssen transparent machen, in welcher Rolle sie kommunizieren und welche Rollenwechsel sie ggf. vornehmen. Um die Glaubwürdigkeit der Wissenschaft zu wahren, sollten die Ziele der Kommunikation und ggf. damit verbundene eigene Interessen offengelegt werden, ebenso die Grenzen des eigenen Fachs und der eigenen Expertise." Dies gilt insbesondere für die digitale „Direktkommunikaton", etwa in Netzwerken. Der Wissenschaftsrat stellt weiterhin explizit fest, dass „z. B. die wissentliche Übertreibung von Forschungsergebnissen, das Verschweigen oder das fälschliche Behaupten von Unsicherheiten als wissenschaftliches Fehlverhalten gelten". Er empfiehlt daher „den wissenschaftlichen Einrichtungen, anschließend an die genannten Vorarbeiten auf möglichst breiter Basis Regeln zur Sicherung kommunikativer Integrität zu vereinbaren und in alle Regelwerke zur Sicherung wissenschaftlicher Integrität aufzunehmen".

Der Ethikkodex des Vereins für Socialpolitik greift genau diese Überlegung auf. Im Punkt 6 des Ethikkodex zur Wissenschaftlichen Praxis heißt es: „Bei wirtschaftspolitischer Beratung oder Kommunikation mit den Medien soll sorgfältig auf den Unterschied zwischen Tatsachenbeschreibung und wissenschaftlich gestützter Aussage einerseits und Werturteil andererseits geachtet werden" (Verein für Socialpolitik, 2021). Digitale Medien werden nicht ausdrücklich genannt, aber Punkt 5 des Kodex hebt mit der Nennung von nicht wissenschaftlichen Medien auch ein Problem digitaler Kommunikation hervor. „In wissenschaftlichen Arbeiten sollen Sachverhalte benannt werden, die potenziell zu Interessenkonflikten oder Befangenheit der verfassenden Personen führen könnten. Diese Regel soll nach Möglichkeit auch bei Veröffentlichungen in nicht wissenschaftlichen Medien angewandt werden."

Die Expert:innengruppe „FactoryWisskomm", die vom BMBF zusammengerufen wurde, um aus der Praxis heraus eine Art Leitlinie zur Wissenschaftskommunikation zu erarbeiten, hängt dieses Thema zu Recht hoch (FactoryWissKomm, 2021): „Die #FactoryWisskomm betrachtet verantwortungsvolle Wissenschaftskommunikation als integrales Element des Wissenschaftssystems. Dabei unterliegt Wissenschaftskommunikation den gleichen Erwartungen und Standards, die an gute Forschung und Lehre angelegt werden: Sie ist integer in ihren Inhalten und Methoden, sie ist relevant, nachvollziehbar, verständlich und transparent. Sie ist forschungsbasiert und reflektiert entsprechend auch ihre Gelingensbedingungen und ihre Folgen für Gesellschaft und Wissenschaft. Wissenschaftskommunikation setzt auf Austausch und Dialog, auch im Sinne des Public Engagements. Sie vermittelt nicht nur Ergebnisse und Methoden der Wissenschaft, sondern hilft auch dabei, gesellschaftliche Problem- und Fragestellungen ins Wissenschaftssystem hineinzutragen. Sie differenziert zwischen Wissensvermittlungs-, Dialog-, Public-Engagement-und Public-Relations-Formaten. (…) Wegen der hohen Verantwortung sollten ethische Grundsätze der Wissenschaftskommunikation gelehrt und gelebt werden." Weiter heißt es: „Die Beiträge verschiedener

Expert:innen und wissenschaftlicher Disziplinen zur Lösung komplexer Probleme sind mitunter widersprüchlich und müssen gerade deswegen transparent und nachvollziehbar kommuniziert werden. Damit die Menschen diese Informationen einordnen und bewerten können, ist es wichtig, die Arbeitsweise von Forschung zu verdeutlichen, die nicht endgültige Wahrheiten erzielen kann, sondern ihre Stärke vor allem aus ihrem inhärenten Problembewusstsein und Skeptizismus zieht. Wissenschaftskommunikation wahrt die Distanz zu persönlichen Werturteilen. Gute Wissenschaftskommunikation bedeutet, dass unter Umständen weniger statt mehr kommuniziert wird."

Weingart et al. (2022) fassen schließlich die Ergebnisse der einschlägigen Arbeitsgruppe der Berlin-Brandenburgischen Akademie der Wissenschaften (BBAW) wie folgt zusammen – und bieten damit die Basis für den folgenden Abschnitt dieses Beitrags: „Da die Verantwortung für direkte Wissenschaftskommunikation nur bei den einzelnen Wissenschaftlern liegen kann, ist es umso wichtiger, dass eine ‚Ethik der Wissenschaftskommunikation' entwickelt wird. Diese sollte zusammen mit oder als Teil der Regeln guter wissenschaftlicher Praxis im Studium und hier vor allem in der postgradualen Ausbildung vermittelt werden. Die Formulierung entsprechender Leitlinien und Grundsätze könnte beispielsweise die Aufgabe der wissenschaftlichen Fachgesellschaften sein. (…) Ebenso bedarf es der Regeln für alle Formen der institutionellen Kommunikation (Public Relations, Werbung, Marketing) und geeigneter Instrumente zur Sicherung ihrer Qualität. Die primär auf wissenschaftliche Information und Vermittlung wissenschaftlicher Fakten ausgerichteten Formen der institutionellen Kommunikation sollten der Kontrolle des Senats einer Universität oder vergleichbaren akademischen Gremien (bzw. Kuratorien von außeruniversitären Forschungseinrichtungen) unterliegen und möglichst vom reinen Wissenschaftsmarketing der Institution getrennt sein."

## Schlussfolgerung für wirtschaftswissenschaftliche Wissenschaftskommunikation

Um die Politikberatung in Deutschland zu verbessern, die ja nicht nur eine Beratung von Politiker:innen ist, sondern in Form von Wissenschaftskommunikation eine „Beratung" der gesamten Öffentlichkeit, sind weniger Reformen der die Politik beratenden Gremien notwendig, sondern strukturelle Änderungen im Wissenschaftssystem, die dazu führen, dass Forschende freiwillig – aus ethischen Gründen – der Maxime folgen „Du sollst nicht übertreiben" (vgl. auch Wagner, 2017). Dies sollte die Ansätze einer Ethik der Politikberatung (als Teil der Forschungsethik) zügig praxisrelevant werden lassen. Dabei geht es darum, dass Wissenschaftler:innen mit größerer Disziplin als bislang der Politik und der Öffentlichkeit deutlich machen, welche ihrer Aussagen und Empfehlungen auf wissenschaftlichen Erkenntnissen beruhen (theoretischer und empirischer Art) und welche (reine) Meinungsäußerungen darstellen. Wenn der wissenschaftliche Stellenwert der Äußerungen von Wissenschaftler:innen besser für Politik und Öffentlichkeit erkennbar ist als bislang, werden wissenschaftliche Forschungsergebnisse eine bessere Rolle im politischen Entscheidungsprozess spielen können als bislang. Jahrzehntelange Erfahrungen zeigen mir: Die Größe von Schlagzeilen, die einzelne meinungsstarke Forschende haben, spielt keine Rolle für erfolgreiche forschungsbasierte wissenschaftliche Politikberatung.

Neben diskussionswürdigen Umbauten der institutionalisierten Politikberatung sind vor allem bessere Strukturen im Wissenschaftssystem selbst notwendig. Mögliche institutionelle Um- oder Neubauten der Beratung selbst seien hier nur stichwortartig genannt: etwa direkte Beratung durch hochrangige Forschende innerhalb von Ministerien (durch auf Zeit berufene „Chef-Wissenschaftler:innen"), aber auch eine verbesserte wissenschaftliche Beratung der Oppositionsparteien (etwa innerhalb ihrer Stiftungen, Wagner, 2005) oder der Umbau des von Interessenvertretungen dominierten Sozialbeirats der Bundesregierung zu einem wissenschaftlichen Beratungsgremium (Wagner, 2021). Entscheidend aber sind strukturelle Änderungen im Wissenschaftssystem, die der oben erwähnten Transparenz bezüglich wissenschaftlicher Ergebnisse und Meinungsäußerungen dienen sowie dafür sorgen, dass einzelne Wissenschaftler:innen ihre Forschungsergebnisse übertreiben und zudem noch Werturteile als Wissenschaft verkaufen. Damit dieses Unterfangen nicht auf eine Zensur Einzelner hinausläuft (etwa durch eine Nationale Akademie; vgl. auch Borges 2022) ist das Ziel der Nichtübertreibung nur durch das verinnerlichen einer entsprechenden Forschungs- bzw. Kommunikationsethik erreichbar.

Langfristig am wichtigsten ist das nachhaltige verankern einer Ethik der Politikberatung im Wissenschaftssystem, und dort insbesondere im Bereich des wissenschaftlichen Nachwuchses (weil der Nachwuchs automatisch in einflussreiche Positionen hineinwächst, ist er als Zielgruppe so wichtig). Mit den Leitlinien des Vereins für Socialpolitik (2021) sind wir auf einem guten Weg. In vielen Doktorandenprogrammen wird auch – mehr oder weniger explizit – die Ethik der Politikberatung gelehrt. Die Ethik der Politikberatung ist so wichtig wie nie, da sich Wissenschaftler:innen im Zeitalter der Digitalisierung direkt an das breite (oder auch spezielle) Publikum wenden können, ohne eine dazwischengeschaltete professionelle Kontrolle durch Journalist:innen, die in der Vergangenheit die Verbreitung groben Unfugs verhindert haben (aber auch neue Ideen durchaus auch mal unterdrückt haben). Die durch die Digitalisierung möglich gewordene freie Meinungsäußerung ist ohne Zweifel zu begrüßen, sie erfordert aber auch – wie alle Freiheiten – Disziplin, also hier eine gelebte Ethik der Politikberatung bzw. der Wissenschaftskommunikation im Sinne von „Du sollst nicht übertreiben".

Neben der individuellen Ethik der Politikberatung können auch institutionelle Strukturen helfen, Politik und Öffentlichkeit deutlich zu machen, welchen Stellenwert die Äußerungen von Hochschulen, Forschungsinstituten und einzelnen Wissenschaftler:innen haben (Wormer, 2022). So sollte in Hochschulen und Forschungsinstituten Wissenschafts-PR und Wissenschaftskommunikation endlich organisatorisch getrennt werden und die Wissenschaftskommunikation auf keinen Fall der Leitung von Einrichtungen direkt unterstellt werden. Dies wurde bereits 2014 von der Leopoldina empfohlen und ist sicherlich durch die digitale Direktkommunikation wichtiger geworden denn je: Die direkte Verbreitung von Privatmeinungen öffentlicher Intellektueller ist nicht Aufgabe von Forschungseinrichtungen und trägt im Übrigen zur Verwirrung bei, was gesicherte wissenschaftliche Evidenz und was Meinungsäußerung ist.

**Title:** ***Self-Discipline Makes Policy Advice Better***

There is a differentiated literature on policy advice, but it is largely disconnected from the literature (and the underlying research landscape) on academic communication, which originally dealt only with the natural sciences. And both strands of the discussion are barely connected to the figure of the „public intellectual“ who has played an important role in the humanities and social sciences for decades. This is a role that, as discussed in this article, is now played digitally by many scholars with low barriers to entry. This creates new problems, which can be addressed by research ethics. Of course, the relevant literature in communication studies is not discussed in detail in this article, but it is pointed out that (selective) examination of this literature might also be worthwhile with regard to policy advice.

Gert G. Wagner ist Mitglied im Sachverständigenrat für Verbraucherfragen (SVRV) und im Sozialbeirat der Bundesregierung.

The original online version of this article was revised due to incorrect page number.

Change history

9/12/2022

Zu diesem Beitrag wurde ein Erratum veröffentlicht: 10.1007/s10273-022-3263-8


Literatur

Bender, K. (1972), Vossische Zeitung (1617–1934), in H.-D. Fischer (Hrsg.), Deutsche Zeitungen des 17. bis 20. Jahrhunderts, Verlag Dokumentation, 25–39.
BBAW — Berlin-Brandenburgische Akademie der Wissenschaften (2008), Leitlinien Politikberatung.
Bock H M Der Intellektuelle als Sozialfigur Archiv für Sozialgeschichte 2011 51 591 641
Borges, Michael (2022), Drosten-Vorschlag zu Kommunikation der Wissenschaft: Ein „Mandat“ fürs Sprechen mit Medien?, auf: Deutschlandfunk, 30. März 2022, https://www.deutschlandfunk.de/wissenschaftskommunikation-wissenschaftsjournalismus-corona-drosten-100.html (5. Juli 2022)
Deutsche Akademie der Naturforscher Leopoldina et al. (2014), Zur Gestaltung der Kommunikation zwischen Wissenschaft, Öffentlichkeit und den Medien — Empfehlungen vor dem Hintergrund aktueller Entwicklungen.
Deutsche Akademie der Naturforscher Leopoldina et al. (2017), Social Media und digitale Wissenschaftskommunikation (2017) — Analyse und Empfehlungen zum Umgang mit Chancen und Risiken in der Demokratie.
FactoryWisskomm (2021), Handlungsperspektiven für die Wissenschaftskommunikation, https://www.bmbf.de/bmbf/shareddocs/downloads/files/factory_wisskomm_publikation.pdf?__blob=publicationFile&v=2 (30. Juni 2022).
Fratzscher M Wagner G G Realistische Erwartungen und ein Blick über die Grenzen tun gut Wirtschaftsdienst 2013 93 8 520 522
Haucap J Thomas T Wagner G G Welchen Einfluss haben Wissenschaftler auf Medien und die Wirtschaftspolitik? Wirtschaftsdienst 2015 95 1 68 75 10.1007/s10273-015-1780-4
Hoffmann L Wagner G G Die Rolle der empirischen Wirtschaftsforschung für die Politikberatung Wirtschaftsdienst 1998 78 3 185 192
Leif, T. (2015), Mythos Politikberatung: Das Schattenmanagement der Lobbyisten, in P. Weingart und G. G. Wagner (Hrsg.), Wissenschaftliche Politikberatung im Praxistest, 229–247.
Müller-Jung J Corona und der Kampf gegen Lügen Frankfurter Allgemeine Sonntagszeitung 2021 49 10
Verein für Socialpolitik (2021), Ethikkodex des Vereins für Socialpolitik (Fassung Dezember 2021).
Wagner, G. G. (2005), Die Rolle der Wissenschaft muss in der Politikberatung klar erkennbar sein — Ein Diskussionsbeitrag, der auch die Opposition nicht vergisst, in D. Haubner, E. Mezger und H. Schwengel (Hrsg.), Agendasetting und Reformpolitik — Strategische Kommunikation zwischen verschiedenen politischen Welten, 375–397.
Wagner G G Volkswirtschaftslehre und Politikberatung Wirtschaftsdienst 2006 86 1 19 22
Wagner G G Effektive Politikberatung Wirtschaftsdienst 2011 91 3 151 152 10.1007/s10273-011-1198-6
Wagner, G. G. (2015), Welche Rolle kann wissenschaftliche Evidenz in der (wissenschaftlichen) Politikberatung sinnvollerweise spielen?, in P. Weingart und G. G. Wagner (Hrsg.), Wissenschaftliche Politikberatung im Praxistest, 189–216.
Wagner, G. G. (2017), Ethische Prinzipien beim Forschungsprozess und dessen Verwertung sind nur durch Selbstdisziplin der Akteure durchsetzbar, Forschung, Heft 3 und 4, 83–90.
Wagner, G. G. (2021), Die Ideen der Rentenkommission in Ruhe diskutieren, Frankfurter Allgemeine Zeitung, 11. April 2020, 24.
Weingart, P., H. Wormer, T. Schildhauer, B. Fähnrich, O. Jarren, C. Neuberger, J.-H. Passoth und G. G. Wagner (2022), Gute Wissenschaftskommunikation in der digitalen Welt — Politische, ökonomische, technische und regulatorische Rahmenbedingungen ihrer Qualitätssicherung, Wissenschaftspolitik im Dialog: Eine Schriftenreihe der Berlin-Brandenburgischen Akademie der Wissenschaften, Nr. 19.
Wissenschaftsrat (2021), Wissenschaftskommunikation (Positionspapier).
Wormer H Von der Wissenschaftskommunikation zur evidenzbasierten Information Aus Politik und Zeitgeschichte 2022 26–27 42 48
